# Disability barriers autistic girls face in secondary education: A systematic review

**DOI:** 10.1177/13623613241294189

**Published:** 2024-11-12

**Authors:** Kathryn Urbaniak, Miranda D’Amico

**Affiliations:** Concordia University, Canada

**Keywords:** autism, girls, lived experience, school experience, secondary education, women

## Abstract

Autistic adolescent girls face complex and diverse challenges in the school setting, specifically mental health issues, unmet social and education needs, and social exclusion. This review provides an overview of research relating to the barriers autistic females face in school settings by reporting on their experiences as well as those of autistic women reflecting on their past. This literature review presents peer-reviewed journal articles published from January 2013 to March 2023. Based on the 17 identified articles, the barriers girls face in the compulsory education setting centred on 4 themes of societal barriers grounded in gender; the institutional or physical barriers of schools; social and communicative expectations; and stigmatization. These results underscore the need for future research to centre diverse autistic lived experiences and knowledge, and autistic inclusion at all levels of participation, from self-advocacy and peer support spaces to the co-development of training and policy.

**Lay abstract:**

Autistic adolescent girls face complex and diverse challenges in the school setting, specifically mental health issues, unmet social and education needs, and social exclusion. The purpose of this review was to provide a general idea of research relating to the experiences of autistic females in secondary school settings by reporting on their experiences and the lived experiences of autistic women reflecting on their past. Based on the identified articles, the barriers girls face in the compulsory education setting centred on four themes of societal barriers grounded in gender; the institutional or physical barriers of schools; social and communicative expectations; and stigmatization. The study highlighted that there is a need to sensitize and educate widely on the topic of autism for teachers, to support staff, school psychologists and peers of autistic youth. The results call attention to the need for future research to focus on the different lived experiences and knowledge of autistic girls.

## Introduction

Autism spectrum disorder (ASD; hereafter, ‘autism’) is a complex, lifelong neurodevelopmental disorder or condition that affects social reciprocity, communication and patterns of repetitive behaviour. Autism is largely diagnosed in childhood although the widening of criteria may have led to an increase in adult diagnosis (Happé et al., 2016). Autism exists without diagnosis; however, clinical diagnosis is generally required to access services including financial aid and special education services. The assessment is conducted behaviourally and is based on observation and reporting of the core characteristics that impact everyday functioning (American Psychiatric Association, 2013). In this article, we adopt identity-first language (e.g. ‘autistic person’) to align with the preferences of many in the autistic community (Botha et al., 2021), except when directly quoting studies.

More boys than girls are diagnosed as autistic at a rate of approximately 4:1 (Loomes et al., 2017). The disparity may be due to several factors, one of which is argued to be that the ‘classic’ characteristics or traits of autism in males differ from those in females. For example, autistic females demonstrate less overt special interests (Frazier et al., 2014), or interests that are deemed more socially acceptable and relational-focused rather than technically-focused (Grove et al., 2018; Nowell et al., 2019). In addition, gender-focused activities and interactions may further influence the social behaviour of autistic girls (Sedgewick et al., 2019).

Camouflaging or masking autistic traits and difficulties may also be a factor in making diagnosis more difficult in women and girls (Ratto et al., 2018). It leads to concealing behaviours such as stimming or repetitive behaviours, using social scripts to appear non-autistic, or engaging in neurotypical behaviours such as forced eye contact or mimicked facial expressions (Lai et al., 2017). As an adaptive compensation mechanism, it is based on social expectations, particularly the gendered expectations of women and girls (Tubío-Fungueiriño et al., 2021). But this phenomenon or coping strategy makes timely and accurate diagnosis more difficult as the challenges faced by women and girls are concealed (Lai et al., 2015). Consequences of masking and the long-term impact of unmet needs are described across the literature as anxiety, depression, low self-esteem, social isolation and self-harm due to the high demands of the camouflaging and the subsequent internalization of these symptoms, thus resulting in higher levels of mental health disorders (e.g. Baldwin & Costley, 2016; Hull et al., 2017; Tubío-Fungueiriño et al., 2021).

Gender is a factor in how clinicians and educators make decisions in identifying autistic behaviours, and gender stereotypes potentially decrease their sensitivity to identification (Bargiela et al., 2016). Gaps in educational professionals’ confidence in identifying and supporting autistic girls compared to boys are suggested to be due to a lack of both understanding and structural support in the educational setting (Gray et al., 2021). Diagnostic bias may come partly from the diagnosis criteria themselves, which have historically been established on a male phenotype of autism (Hebron & Bond, 2019). Subsequently, the participants enrolled in empirical autism research are those that have received a diagnosis based on ‘classic’ autism and not those who are still being missed (Lai et al., 2015).

Given this diagnostic bias, it is not surprising that autistic girls are diagnosed later than boys (Begeer et al., 2013), meaning they miss out on various supports in childhood and into adolescence. Challenges with social behaviour become more apparent for girls as childhood strategies, such as imitation, are no longer enough for the complexities of adolescent same-sex friendships (Bauminger et al., 2008). Autistic girls seem to be more prone to experience internalising symptoms and depression or anxiety than boys who exhibit more hyperactive traits, potentially contributing to the underdiagnoses of girls (May et al., 2014). In fact, girls with equivalent levels of autistic characteristics to boys still need to demonstrate more difficulties in order to receive an autism diagnosis (Duvekot et al., 2017; Dworzynski et al., 2012).

Another identity dimension that results in under- and over-diagnosis is race/ethnicity, potentially from implicit bias. For example, Golson et al. (2022) found school psychologists did not fully consider cultural and linguistic factors when considering special education classification. Rather they focused on behaviours identified as problematic leading to racial/ethnic disparity in addition to gender disparity. It is also important to note that the binary approach used in this review is a product of the literature available; however, it excludes a substantial minority of transgender, non-binary and gender-fluid autistic people (Dewinter et al., 2017; Warrier et al., 2020). In addition, binary gender norms in autism research do not well represent transgender individuals’ experiences of living as different genders at different times such as childhood and adulthood (Strang et al., 2018).

Despite the issues with the under- and misdiagnosis of autistic girls, there is an increase overall in the prevalence of autism (Public Health Agency of Canada, 2018). The increase may be attributed to the widening of criteria under the umbrella of autism spectrum disorder and increased knowledge and awareness. An increase of diagnoses in the general population is reflected in an increased prevalence within school systems in various countries where a number of interventions support autistic children’s special educational needs (Dean & Chang, 2021; Tsou et al., 2024).

When asked what autism research should be focused on, Pellicano et al. (2014) found the autistic community in the United Kingdom identified education and inclusion as priority areas; however, autistic voices are often excluded from research as well as decision making such are where autism funding is allocated.

The complexity of the secondary school setting requires the transition to a new, often bigger and busier, physical environment, in addition to changes in organizational structure such as more subjects, classrooms and different teachers. In addition, this time marks a shift in social relationships in adolescence that create challenges for autistic girls (Morewood et al., 2019). Gender differences in social relationships are well established where girls’ relationships are more socially-driven (Rudolph & Dodson, 2022). The timing of the transition to secondary school often coincides with the onset of puberty. This maturation and changes in expectations around friendship, pose additional challenges (Bargiela et al., 2016; Cook et al., 2018).

Historically autistic voices, particularly female voices, have not been centred in autism research (Hull et al., 2017; Strang et al., 2018). This literature review examines current issues, in particular disabling barriers, autistic women and girls aged 11 years and older face during the time in which they are still legally required to attend school, with an emphasis on amplifying their firsthand views and experiences. The age of 11 years old and up was chosen because it is when most students enter secondary school.

The literature review question was defined as follows: What are the disability barriers girls face in secondary education?

## Methods

This qualitative literature review situates autism in the social model of disability meaning it examines the disabling barriers generated by society that autistic individuals face rather than focusing on impairment through the medical or deficit model of disability (Oliver, 2013).

To identify the included studies, a systematic search strategy was employed. Peer-reviewed journal articles in English from January 2013 to March 2023 were obtained from Scopus, PubMed and PsycINFO as well as Academic Search Complete, Education Source, and Educational Resources Information Center (ERIC) via EBSCO. These databases were chosen to ensure a breadth of education research could be gathered.

The following keyword combinations were employed: autism OR asd OR autism spectrum disorder OR Asperger’s OR Asperger’s syndrome OR autistic disorder OR Asperger’s AND education OR school OR learning OR teaching OR classroom OR education system OR educational AND women OR woman OR female* OR girl*. The following pre-defined inclusion criteria were used:

1. Studies included women and girls with a diagnosis of autism reflecting on their secondary school educational experiences.
2. Studies focused on the experiences of autistic women and girls including their narrative accounts, not solely observations by parents and/or educators.
3. Studies focused explicitly on lived experiences where women and girls’ experiences were expressed separately from men and boys’ experiences.
4. Papers written in English.
5. Papers published in a peer-reviewed journal.

**Figure 1.**
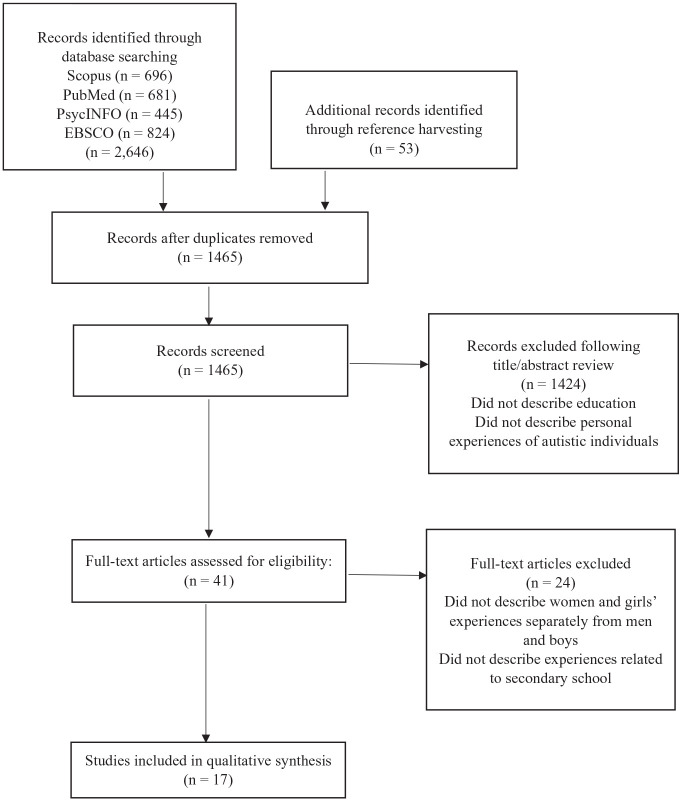
Flow diagram showing study selection.

The initial search yielded 2646 records (Figure 1). The first author screened all search results by a review of abstracts. Duplicate records were removed. Studies were discarded if they were biomedical research rather than education research. In addition, studies were excluded if they focused solely on preschool, college and university settings. The remaining 41 articles were read in full by the first author. The studies excluded were those that did not explicitly separate the experiences of women and girls. Then the remaining 17 selected studies were closely read several times by both authors. We conducted a thematic synthesis of the findings, discussions, and recommendations in each article. This included the first-order constructs (the reported responses from the autistic participants) and second-order constructs (parents’ experiences and observations, and researcher interpretations). The experiences and insights provided by the women and girls themselves were prioritized as they were the focus of this review and comprised the majority of the data. The details drawn from this examination were collated in tables and categorized by first conducting elemental structural coding of the texts, followed by second cycle coding for similarity and frequency leading to the thematic analysis to identify the major themes (Saldaña, 2013). To address validity, the authors discussed each of the 17 reviewed papers to achieve consensus. Our interpretation used a reflexive approach to provide reviewer insight into the topic of autistic lived experience. The authors acknowledge the positionality of the review, led by a female autistic scholar. *Community involvement*: The first author is an autistic scholar; this literature was reviewed through the autistic lens.

## Findings

### Overview of the included studies

As illustrated in Table 1, the 17 studies were conducted in various English-speaking countries. Three were conducted with participants UK-wide (Bargiela et al., 2016; Honeybourne, 2015; Tierney et al., 2016), seven in England (Cook et al., 2018; Essex & Melham, 2019; Halsall et al., 2021; Myles et al., 2019; Rainsberry, 2017; Sproston et al., 2017; Tomlinson et al., 2022), one in Northern Ireland (Goodall & MacKenzie, 2019), one in Ireland (Ryan et al., 2021), four in Australia (Baldwin & Costley, 2016; Cridland et al., 2014; Jarman & Rayner, 2015; Vine Foggo & Webster, 2017) and one in the United States (Matthews et al., 2019).

**Table 1.** Characteristics of selected studies.

Author(s)	Location	Description of participants as labelled by the authors	Description of methodology	Description of themes
Baldwin and Costley (2016)	Australia	82 women with high-functioning ASD Age 18–64 Part of a larger mixed study	Survey Descriptive, statistical and thematic analysis	Diagnosis and ASD Symptomatology; health and mental health; education; unemployment; social experiences; general support needs
Bargiela et al. (2016)	UK	14 women diagnosed with ASC in late adolescence or adulthood Age 19–30	Semi-structured interviews Framework Analysis	‘You’re not autistic’; pretending to be normal; passive to assertive; forging an identity as a woman with ASD
Cook et al. (2018)	England	11 girls with autism Age 11–17 years 11 parents	Semi-structured interviews Thematic analysis	Motivated to have friends; challenges faced by girls with autism; masking their autism: both a solution and a problem
Cridland et al. (2014)	Australia	3 girls with ASD Age 12–17 5 mothers	Semi-structured interviews Interpretative phenomenological analysis	Diagnostic issues; being surrounded by boys; experiences of high school; complexity of adolescent female relationships; puberty; sexual relationships; impact of having an adolescent daughter with ASD
Essex and Melham (2019)	England	4 young women with high-functioning autism Age not reported 4 education professionals	Semi-structured interviews Phenomenological psychological approach	Inconsistent and frequently inadequate management of educational transition; successful transition relies too heavily on individuals, rather than on consistent processes; curriculum preferences that defy stereotypes
Goodall and MacKenzie (2019)	Northern Ireland	2 teenage girls with Asperger’s Syndrome (AS) Age 16 and 17 Part of a larger mixed study	Semi-structured interviews Drawing activity Inductive thematic analysis	Impact on well-being; bullying and friendships; inclusion
Halsall et al. (2021)	England	8 autistic adolescent girls Age 12–15 8 mothers 7 educators	Semi-structured interviews Reflexive thematic analysis Social constructionist perspective	Inconsistencies, contradictions and conflicts in attempts to camouflage; using camouflaging to overcome challenges in making and maintaining friends; camouflaging learning needs and the challenges of learning and inclusion; consequences of camouflaging on social interaction, learning and mental health
Honeybourne (2015)	UK	67 women and girls on the autism spectrum Age 14–50 years	Survey. Open interviews General thematic analysis	The positives; difficulties and suggestions for schools; friendships; communication; learning; interpreting the world of school; feeling (mis)understood (Note: a priori not emerging themes)
Jarman and Rayner (2015)	Australia	30 women with AS 15 parents of girls with AS currently attending school	Survey Inductive thematic analysis	Teachers’ recognition of the AS diagnosis in females; lack of understanding about the challenges associated with AS; helpful attitudes and actions of teachers
Matthews et al. (2019)	USA	4 girls with ASD Age 16–21	Semi-structured interviews Guided journal activities	Emotions around transition process; own involvement in planning; other people and levels of planning; own dreams for future
Myles et al. (2019)	England	8 girls with autism Age 12–17	Semi-structured interviews	Reciprocal friendships; feeling safe and supported; encouragement and inclusion; establishing and adhering to social expectations; being on the periphery; feeling devalued
Rainsberry (2017)	England	3 girls with autism Age 13–16	Semi-structured interviews Creation of comic books	Special friendships; friendships and peers; special interests; teachers and school staff; attitudes of staff; subject lessons; routine, structure and support; diagnosis and disclosure; secondary school versus primary school
Ryan et al. (2021)	Ireland	10 girls with AS, ASD or childhood autism diagnosis Age 12–15	Focus groups (2) Inductive thematic analysis	Friendship; friendship volatility; friendships and technology; gender differences
Sproston et al. (2017)	England	8 autistic girls Age 12–17 8 parents	Semi-structured interviews Inductive thematic analysis	Inappropriate school environments; Tensions in school relationships; Problems with staff responses
Tierney et al. (2016)	UK	10 girls with ASC Age 13–16 Parents	Semi-structured interviews Inductive thematic analysis	Experiences of social environment; desire for friendships; overcoming challenges; developmental tasks
Tomlinson et al. (2022)	England	3 autistic girls Age 14–16 3 mothers 1 support staff	Semi-structured interviews Inductive thematic analysis	School environment; social interactions; self-identity; personal challenges; facilitators; awareness of autism/individual need
Vine Foggo and Webster (2017)	Australia	7 girls with ASD or AS Age 13–17	Semi-structured interviews – verbal or written Inductive thematic analysis	Social interactions are rewarding but difficult;

The studies were either qualitative or mixed methods. Thirteen studies utilized semi-structured interviews, three used surveys, and one employed focus groups. Participant sample sizes ranged from 2 autistic girls to 82 autistic women and girls ranging from 11 to 64 years old. Three studies reported on race, ethnicity or cultural background (Baldwin & Costley, 2016; Cook et al., 2018; Halsall et al., 2021). The remaining studies did not report on race in any way. Seven studies included parents’ voices, and three studies included educators’ voices. Adolescent and young adult experiences were the primary focus of 13 studies and 4 studies considered the experiences of adult autistic females reflecting on their past schooling.

Adolescent lived experience studies specific to school were conducted by Goodall and MacKenzie (2019), Rainsberry (2017) and Tomlinson et al. (2022). In contrast, Honeybourne (2015) and Jarman and Rayner (2015) reported on adult retrospections on experiences in school. More general experiences that provided insight into school as one of multiple areas were investigated by Cridland et al. (2014) with adolescents, and by Baldwin and Costley (2016) and Bargiela et al. (2016) with adults. The remaining studies examined specific areas of the adolescent school experiences of autistic girls. Sproston et al. (2017) reported on experiences of both temporary and permanent school exclusion. Myles et al. (2019) investigated social experiences and the sense of belonging in schools. Both Halsall et al. (2021) and Tierney et al. (2016) addressed camouflaging/masking. In addition, Cook et al. (2018), Ryan et al. (2021) and Vine Foggo and Webster (2017) investigated lived experiences around the theme of friendship. Finally, Essex and Melham (2019) and Matthews et al. (2019) reported on the transition from secondary school to vocational and further education or the workplace.

The language choices made by the authors to describe autism varied based on geographical location, time and their stance (see Table 1). Both identity-first and person-first language were used in the studies. Autism was primarily described as a ‘disorder’ (e.g. Autism Spectrum Disorder); however, two of the studies (Bargiela et al., 2016; Tomlinson et al., 2022) used the word ‘condition’ (e.g. Autism Spectrum Condition). Language choice has broad implications for policy and research agendas. How the language describing autism is used is an ongoing conversation, particularly within the growing field of critical autism research where identity-first language reflects a move for autonomy by autistic individuals (Botha et al., 2021); however, this is outside the scope of this article and potential for future research.

The following section reports on the barriers and recommendations given by the participants in the primary studies, presented as four themes. First are the societal barriers grounded in gender, which include historically male-centric diagnostic instruments, biases in diagnosis and gendered stereotypes. Second, there are institutional or physical barriers generated by insensitive and inattentive school environments. Third are the social and communication expectations of the neuro-majority, such as the social reciprocity, with which autistic girls often struggling with in making and maintaining friendships. The last theme is stigmatization leading to masking, which is a barrier to neuro-affirming identity formation and positive mental health.

### Societal barriers grounded in gender

The theme of social barriers grounded in gender in the literature was expressed as categories of disbelief, isolation, and gendered expectations and demands. The assumptions of educators and professionals that girls cannot be autistic still exist and autistic girls can be met with responses as strong as incredulity, such as a principal laughing at the idea of a girl with autism as exampled by Jarman and Rayner (2015). Participants in Cridland et al.’s (2014) study described being surrounded by boys as isolating as well as services and activities being catered more to boys which furthered feelings of not fitting in.

Autistic girls also reported gendered social behaviours as barriers when describing peer social interactions. They described themselves as ‘ungirly’ in terms of their interests or appearances, making it harder to fit in (Tierney et al., 2016). In Baldwin and Costley’s (2016) study, significantly more of the female participants described being under-supported socially at school; their teachers were under-informed and under-educated on autistic girls, and did not flag or notice their support needs. Similar findings were reported by the autistic girls participating in a study by Halsall et al. (2021). Participants reported that being quiet and passive was seen as more socially acceptable, but this contributed to not getting the support they needed nor meeting their academic potential (Bargiela et al., 2016). A participant in the study by Essex and Melham (2019) described wanting staff to know that if she was sitting quietly and not working, that she actually needed help but was unable to ask. Others described experiencing performance anxiety of being judged if they raised their hand in class (Sproston et al., 2017).

Girls in Rainberry’s study (2017) reported teachers who were overly negative and described them as lazy and rude. They were also branded as disorganized and messy and held to higher standards than the boys in their classes (Baldwin & Costley, 2016; Honeybourne, 2015). The girls in the study by Halsall et al. (2021) reported hiding both their autistic traits and their learning challenges; this led to missed learning and under-achievement, as well as to the educators being unaware of the girls’ academic concerns as the inclusive class demands increased. Parent participants also described that when their daughters masked all day, they then had a meltdown at home; this means that the teachers only saw a façade that took the participant a great deal of effort to maintain during the school day (Jarman & Rayner, 2015).

The challenges rooted in gender biases include delayed diagnoses, insufficient support and pervasive stereotypes, thus impacting the educational achievements and social integration of autistic girls. A number of recommendations were included in the studies and are presented in the discussion section of this article.

### Institutional or physical barriers

The theme of institutional and physical barriers included environmental challenges such as the sensory overload and the structure of the school day, as well as a range of transitional challenges and challenges with teachers and other staff.

The physical environment of school has been described as a disadvantaging barrier that applies to both autistic girls and boys. However, given the prevalence of masking in girls, some of these problems go unaddressed because people assume they are coping when they are not. Sproston et al. (2017) examined the experiences of autistic girls in inclusive schooling, particularly their temporary or permanent exclusion due to their behaviour. Insensitive and inattentive school environments were identified as one of the major themes leading to their school disengagement, including the difficult sensory experiences, which attributed to the girls’ challenges. Many study participants reported difficulties managing the school environment due to noise leading to frustration and difficulty coping with their own emotions (Cook et al., 2018; Essex & Melham, 2019; Tierney et al., 2016). For example, one participant described not eating at school because the lunchroom was too chaotic (Goodall & MacKenzie, 2019). Another told of the sensory challenge of crowded school corridors given she was sensitive to touch (Tomlinson et al., 2022).

In addition, the structure of the school day added tensions, including changes in locations, shifting teacher expectations, the length of the school day, and an overall lack of agency and control. Educational transition was described as being a challenging process for autistic students given the organizational changes required; this is in addition to changes such as expectations from different teachers, timetabling and materials, unstructured free time, sensory overload and the use of multiple locations (Essex & Melham, 2019).

Transitions are difficult for most autistic individuals. The move from unstructured time, such as recreation and lunchtime, back to structured time was reported as a challenge (Honeybourne, 2015). These changes can be overwhelming and a lack of understanding or support from school staff about the seriousness of day-to-day functioning increases feelings of being overwhelmed (Cridland et al., 2014; Jarman & Rayner, 2015). The movement between different learning environments was also reported as challenging. For example, changing from a segregated special education support unit to an inclusive classroom was described as difficult because the participants need to shift their masking behaviours for different learning environments (Halsall et al., 2021).

The transition to secondary school was described by mothers as challenging for their daughters in terms of moving between classes, organising materials and equipment, and coping with different teachers and classes (Cridland et al., 2014). The support provided during the transitions to the final year of secondary school or further education was described as being reactive and academic rather than holistic and well-prepared, where coordination often became the role of a parent, generally the mother, rather than a support specialist (Essex & Melham, 2019). Participants planning their transition from secondary, or vocational school also described planning meetings as a challenge where they were not comfortable speaking, did not feel heard when they shared their ideas (Matthews et al., 2019). In addition, they found the social dynamics of the meetings a barrier given the number of people involved resulting in them losing their voice in the planning of their own futures.

In the later years of secondary school, career guidance can also be a point of disconnect. For example, gendered assumptions about abilities towards specific subjects like science over the arts is problematic when giving career advice to autistic girls. Three of the four participants in the study by Essex and Melham (2019), intended to study or work in the arts rather than follow stereotypical roles involving math or science. In addition, participants in the study by Matthews et al. (2019), described pressure from, or misalignment with, the career counsellors’ planning, resulting in them ultimately losing their voice.

The boundary around the roles of teachers in schools was also identified as a barrier where some teachers were perceived as abdicating themselves of responsibility for accommodations or individualizations, leaving this to the specialist staff (Tomlinson et al., 2022). Participants also reported anxiety around evaluations where teachers’ uniform approaches to test and exam preparation did not provide enough support. The pressure around high expectations on students to do well in exams also came from teachers (Tomlinson et al., 2022). Individualized approaches to learning, such as Universal Design for Learning (UDL), may address some of these issues for autistic youth, but also for un-diagnosed individuals and other learners with diverse needs.

Recommendations focused on teacher training to include classroom management, social support and awareness-raising. Specific examples included how even if an autistic girl is performing satisfactorily academically, teachers should not assume there are no difficulties or that additional assistance is not required; explanations, help with organization and extra time may still be needed. Teachers should also enquire about the time spent on homework and assignments as difficulties may only be expressed in the home setting (Jarman & Rayner, 2015). Other recommendations from the literature propose educational establishments be proactive in developing inclusive environments (Sproston et al., 2017), including designated quiet places and organized activities at break and lunch times, social coaching and the option to not participate were also recommended (Honeybourne, 2015). Organization skills, for example, around time management, workload and organization of materials are needed (Jarman & Rayner, 2015), with more systematic and holistic transition preparations to better prepare students. An approach that includes social, organizational, and employment elements would benefit those who leave school before graduation in particular (Essex & Melham, 2019). Finally, planning processes that ensure that the youth are able to communicate and are being heard rather than ignored ensures much-needed agency as they move into adulthood (Matthews et al., 2019).

### Social and communication expectations

Social and communicative differences are the primary traits of autism. The theme of social and communication expectations arose in the literature as a barrier where the social expectations of the neuro-majority prevail. Complexity and conflict in relationships with peers and school professionals were prominent categories in the literature along with hard to meet expectations. Young women described their teenage female peers’ social abilities as more sophisticated or complex (Bargiela et al., 2016; Cook et al., 2018). The complexity of female relationships was also increasingly challenging in adolescence and expectations of friendship seemed to diverge between the autistic girls and their neurotypical peers (Cook et al., 2018). Girls described struggling with ‘finding a tribe’ (Halsall et al., 2021). They expressed a desire for friendships but found them exhausting (Vine Foggo & Webster, 2017). In addition, multiple studies described the loss of friendships due to transitions to and from secondary school (e.g. Essex & Melham, 2019; Ryan et al., 2021; Vine Foggo & Webster, 2017).

For adult autistic women reflecting on their memories of school, relationships with others were often the defining theme where almost all cases recalled feelings of isolation, loneliness and difference (Honeybourne, 2015). Both adults and adolescents reported feelings of isolation, such as poor mental health outcomes, and perceived bullying was prevalent throughout the studies reviewed (e.g. Cook et al., 2018; Cridland et al., 2014; Essex & Melham, 2019; Honeybourne, 2015; Sproston et al., 2017).

Some girls reported positive friendships, particularly with other girls with similar neurotypes (e.g. Rainsberry, 2017). However, others described pretending to be like their peers to fit in but committing unknown ‘faux pas’ in secondary school (Bargiela et al., 2016). The unpredictability of peer interaction caused considerable stress and anxiety to some of the girls (Rainsberry, 2017). In some cases, the autistic girls were blamed for the bullying they experienced (Bargiela et al., 2016). While conflict and loss have been described as normal, they are difficult and isolating to experience (Ryan et al., 2021; Vine Foggo & Webster, 2017). In one study, happiness in school was primarily impacted by having friends, particularly those who made the participants feel comfortable, understood and safe given the larger secondary school environments (Myles et al., 2019).

In a study on friendship by Ryan et al. (2021), these connections were found to be very important to the girls. Online friendships were reported as being easier than in real life, but all the girls described preferring to have friends in real life over online friends (Ryan et al., 2021). Participants in another study reported not using social media because it was hard to use with the lack of social cues and the general challenges they face with social communication such as being misunderstood or being perceived as argumentative (Tomlinson et al., 2022).

Social and communication barriers were described as occurring with both peers and with school professionals. Girls who had been temporarily or permanently excluded from school reported tensions with teachers as contributing to their educational experiences including describing challenges with the power differential between teachers and students (Sproston et al., 2017). One participant in Rainberry’s (2019) study referred to her teachers as ‘idiots’, believing that some of her teachers hated her, suggesting significant tensions in those relationships. Some participants experienced tensions with teachers who thought they were being intentionally rude, ‘disrespecting their elders’, or choosing not to listen which was not the case (Myles et al., 2019; Tierney et al., 2016). While some participants reported a lack of support for various reasons, there were also descriptions of tension from too much support, where teaching assistants were too present but the participants themselves did not want attention as they did not want to be seen as different (Tomlinson et al., 2022).

### Stigmatization as a barrier

The topic of masking or camouflaging recurs throughout the autism literature, particularly in reference to women and girls. It is a coping mechanism across many minority groups, done to avoid stigmatization, but has consequences for mental health and identity formation. Masking and mental health, disclosure and community arose under the theme of stigmatization.

Stigmatization preventing parents from making their adolescent children aware of their autism denies their child self-understanding and self-awareness as well as opportunities for community. In the Rainsberry (2017) study, one of the three participants was unaware of her autism diagnosis despite being diagnosed at age 3, and it was unclear whether she was aware of her ADHD diagnosis. In the 2014 study by Cridland et al., five parents participated in interviews for the study, but only three daughters were included as one parent did not give consent for her daughter to participate, and another daughter was not explicitly aware of her diagnosis.

In one study, mothers described their daughters as fitting into neither inclusive settings nor special education settings (Cridland et al., 2014). In another study (Halsall et al., 2021), the girls were camouflaging in both their inclusive and special educational settings, highlighting challenges with relationships and isolation, mental health and learning as they did not ‘belong’ to either group. Interestingly, the educators reported that the girls did not camouflage in the special education context, whereas the girls themselves reported camouflaging in all educational settings. The girls reported hiding both their autistic traits and their learning challenges leading to missed learning opportunities and under-achievement as the educators were unaware of the girls’ concerns.

In Tomlinson et al. (2022), parents and professionals reported a reduction in masking post-diagnosis led to improvements in mental health, potentially due to family and professional support. Participants described being hesitant to disclose to peers fearing the consequences. A young woman in the study by Bargiela et al. (2016) described her diagnosis as a tool that empowered her and gave her more confidence to express herself rather than remain quiet. After spending time in neuro-affirming spaces such as online forums, they also reported increased pride and confidence in their diagnosis. However, in the same study, a participant noted the shift in the language used around her by professionals’ post-diagnosis from ‘when you live alone’ to ‘if you live alone’ demonstrating the need for sensitization and education of professionals.

## Discussion

Overall, this review highlighted literature that centralizes the voices of autistic women and girls in describing disabling barriers in their school experiences. However, a great deal of literature also focuses on the perceptions and observations of professionals without the inclusion of the autistic individuals themselves. Much of the lived experience literature concentrated on the social and communication barriers and masking or camouflaging, which seems in line with current education research when studying autism in women and girls (Tomlinson et al., 2022). The studies included in this review point to an overarching recommendation to widely sensitize and educate teachers, support staff, school psychologists and peers of autistic children on the topic of autism. They indicate a need for a more intersectional and inclusive approach. There is also a clear need for individualized supports in relation to the hidden curriculum, particularly socially, in terms of communication, as well as personalized interventions to prevent exclusion, or withdrawal.

### Societal barriers grounded in gender

The autistic traits of younger girls are generally subtler, but as they age, they struggle more socially as the early strategies like simple imitation no longer work, which can lead to increased anxiety (Corscadden & Casserly, 2021). In their seminal study comparing elementary children with and without a diagnosis of ASD, Dean et al. (2014) established that autistic boys and girls confront different social situations. They found that autistic boys and girls appear more socially comparable and proportionally experienced increased social exclusion than their neurotypical peers. However, they found that autistic boys tended to be visibly socially excluded compared to autistic girls, who tended to be unseen, rather than spurned (Dean et al., 2014). In a more recent study, Dean et al. (2017) highlight how the masking/camouflage premise is manifested in young girls as they navigate the social landscape by staying close at hand to their friends and mingling so as to cover up their social unease. On the other hand, autistic boys typically tended to play alone in the playground thus labelled has having social skills issues. If this is the case, then autistic girls will continue to be underrepresented as their social resourcefulness will continue to be undisclosed (Dean et al., 2017). Many autistic youths have negative experiences of secondary school related to their autism, but autistic girls seem to experience secondary school more negatively than boys (Bottema-Beutel et al., 2020) and may become increasingly solitary (Dean et al., 2023).

The barrier is not their gender but rather the patriarchal systems within which these girls are living and learning. These biases seem to occur especially with individuals, such as teachers, who are not specialized in the assessment but are still gate-keepers to assessment and subsequent supports (Cridland et al., 2014).

The aim is not to blame teachers or individual schools, but rather look critically at the education system, teacher training programmes and places where patriarchal structures can impact behaviours. It is important to acknowledge that masking or camouflaging factors might be the reasons that teachers miss the challenges faced by autistic girls.

Recommendations garnered from the literature proposed awareness-raising of the presentation of autism in women and girls among professionals including clinicians and educators (Cridland et al., 2014). Jarman and Rayner (2015) presented several recommendations for dispelling gender stereotypes and improving training on classroom management and social support for all autistic children. A participant in Goodall and MacKenzie’s study (2019) made recommendations on ways teachers could improve their relationships with autistic students including taking the time to listen and understand the difficulties young autistic people face in school.

Teachers need to be better equipped to prevent missed learning opportunities and under-achievement resulting from masking. They provide students with a central relationship in the schooling experience. The studies reported both supportive and challenging relationships with teachers. This is echoed in the literature by Blacher et al. (2014), in that autistic children tend to have higher levels of conflict in their relationships with their teachers. But positive relationships with teachers are essential as they can provide a stronger sense of well-being and better learning outcomes for autistic students (Caplan et al., 2016). Therefore, it is crucial to ensure teachers are trained and supported to work with autistic children and youth, as well as being sensitized to the breadth of autistic representation and diversity including girls. This training should concentrate on the unique presentations of autism in girls and include the subsequent impact of missed or overlooked diagnoses has on the girls’ education experiences and outcomes.

### Institutional or physical barriers

From sensory overload to unstructured transitions and the reactive nature of support systems, institutional and physical barriers exacerbate both exclusion and disengagement. Interventions need to be tailored to the individual and the situation to support effective positive learning experiences for autistic girls.

### Social and communication expectations

Autistic elementary school girls and boys in secondary school tend to be more social but find it challenging to maintain these interactions, whereas autistic girls in secondary school and boys in elementary school are generally more reserved and less inclined to participate in group activities (Dean et al., 2023). This variation by age and gender underscores the importance of designing social interventions that are customized to build on the existing social strengths of these individuals.

Vine Foggo and Webster (2017) highlighted the need to provide clear and explicit support to negotiate the hidden curriculum of female adolescent social relationships. The findings of Cook et al. (2018) pointed to the need for staff training and programmes to support the social interaction of girls with autism. Given their specific perceptions of friendship, rather than intervening to develop deeper reciprocal friendships, Cook et al. proposed finding opportunities for them to share social spaces based on similar interests, thus potentially reducing the need for masking to fit in while also keeping in mind that addressing issues surrounding bullying is also needed. Other recommendations included additional clinical support for adolescents with mental health problems due to social difficulties and social isolation (Cridland et al., 2014). Finally, Ryan et al. (2021) proposed coaching on the use of texting and social media for safety skills and to enable participants to maintain friendships more successfully.

Problems navigating friendships, and interactions with peers and educators, demonstrate the critical need for tailored support systems. Autistic girls often struggle to make and maintain friendships as they age; therefore, both support and more comprehensive sensitization are needed to ensure inclusion and a sense of belonging. Autistic girls face similar social challenges to autistic boys, but autistic girls experience specific challenges in same-sex friendships with non-autistic peers, particularly with identifying and resolving conflict (Sedgewick et al., 2016). Additional recommendations included providing additional clinical support for adolescents with mental health problems due to social difficulties and social isolation, given the prevalence of mental health challenges for autistic girls (Cridland et al., 2014). While support in navigating the hidden curriculum could be beneficial, it is essential to note that any approach should be individualized based on the wants and needs of the girls as social interactions can be both energising and depleting. Information from autistic girls on how they view and want to maintain their friendships should be central to any interventions.

Another barrier to learning highlighted in the literature was communication challenges within the classroom and in education planning interactions. Such approaches to communication need to be rethought to include multiple modes of communication, and opportunities that are low stakes to ensure equitable participation in learning, and in planning for future learning.

### Stigmatization as a barrier

Alongside increasing staff awareness of autism, especially with girls, it may also be beneficial for schools to incorporate activities to positively promote awareness of differences such as neurodivergences like autism as a mechanism to support adolescents if they wish to disclose their diagnosis to peers (Rainsberry, 2017). The clinical recommendations were awareness-raising of the presentation of autistic females among clinicians, additional clinical support of adolescents for mental health problems due to social difficulties and social isolation, early and individualized sexual education given sexual vulnerability as well as challenges with puberty, physical changes and boundaries, and finally gender-specific social support services (Cridland et al., 2014).

The stigma barrier leads to non-disclosure or hidden identity, but as discussed above, camouflaging has consequences. Therefore, alongside increasing awareness of autism, destigmatization is important.

There are potential benefits to creating supportive, neuro-affirming environments that encourage self-awareness and community engagement. Schools could incorporate activities to positively promote awareness of differences, such as those of neurodivergence like autism, as a mechanism to support adolescents if they wish to disclose their diagnosis to peers (Rainsberry, 2017). This would encourage higher self-esteem and a reduced need for masking.

In addition to removing barriers to learning, barriers to self-advocacy should be considered and challenged. Acceptance, rejection and ambivalence are all reactions to diagnosis from adolescent autistic girls which can impact their sense of self (Gaffney, 2020). In discussing lived experience research, such as the studies explored here, Humphrey and Lewis (2008) present the importance of unpacking how an individual, such as those receiving an autism diagnosis, interact and understand the ‘label’ being attributed to them as part of addressing stigmatization. School-based peer support groups based on shared experiences are a potentially beneficial mechanism that can provide an affirming space for youth to navigate topics such as identity and disclosure. Crompton et al. (2023) suggest that these spaces be inclusive of wider neurodivergence, thus providing individuals with community, inclusion and expertise in addition to indirectly sensitising the wider school community, particularly if led by neurodivergent individuals. While education remains situated in a belief system where autism and other neurodivergence are framed as a deficit, these youth cannot thrive.

Autistic voices, including those of children and youth, should hold equal if not more space in autism education research to challenge the current state of epistemological injustice. Our autistic voices should be centred in gathering recommendations in alignment with the principle of ‘nothing about us without us’. There is an increasing understanding that the lived experience of autistic adults should play a central role in autism research (Woods & Waltz, 2019). In education research, this can be realized by including autistic adults reflecting on their educational experiences where the autistic community, composed of a wide variety of individuals, can be involved in the co-development of planning, policy and training.

The literature presented a wide variety of experiences and recommendations as the institutional and physical environment of secondary school poses multiple barriers for autistic girls. For example, the increased anxiety due to sensory overload in the classroom setting can contribute to academic under-achievement compared to neurotypical students (Ashburner et al., 2010). Many of the studies included recommendations to be proactive in developing inclusive environments. Supports for organization skills around time management, workload and organization of material could also address some of the challenges facing girls in secondary school, with more systematic and holistic transition preparations to better support students. Flexible teaching practices, positive and accepting attitudes and understanding were key elements in the positive experiences of the girls (Jarman & Rayner, 2015).

### Limitations

The limitations of participant diversity and the homogeneity of the studies included in this literature review have already been outlined. Notably, there is an underrepresentation of female perspectives, and minority voices are even further marginalized. In addition, this review was limited to English-only studies across five developed countries. Each of these countries has its own education system and policy as well as different understandings of autism leading to a wide variety of studies. The studies also had ranged in sample sizes and variations in design. While much knowledge about autism is generated outside academia, peer-reviewed journals were selected to focus on the education research being conducted over the past 10 years. Finally, none of the studies reflected the realities of the COVID-19 pandemic, the full impact of which continues to unfold.

### Future directions

The lack of inclusivity in autism research is a primary ethical concern, particularly where members of marginalized groups or communities are systematically excluded (Cascio et al., 2021). An intersectional approach to education research is needed so that policy, training and supports can respond to the needs of underrepresented communities, particularly when research represents individuals with disabilities including autism (García & Ortiz, 2013).

This review focused on the experiences of women and girls. As previously noted, this binary or dichotomous approach to sex/gender is a product of the literature available. This ignores autistic people who identify as transgender, non-binary and gender-fluid (Dewinter et al., 2017; Warrier et al., 2020). Research also suggests that autistic individuals identify as homosexual, bisexual and asexual at increased rates compared to the general population (George & Stokes, 2018). LGBT-identifying autistic youth report more negative experiences with staff and peers in secondary school (Bottema-Beutel et al., 2020). Furthermore, the intersecting identities of LGBT and autism can compound negative experiences such as victimization (Strang et al., 2018). Therefore, given the prevalence of queerness in the autistic community, sexual orientation is an identity group to be considered when conducting research on lived experiences, particularly when describing barriers and belonging.

Parental involvement and advocacy also vary significantly with socioeconomic status, impacting autistic children’s experiences and exacerbating inequalities. Future research should thus consider socioeconomic factors and include diverse parental perspectives, including those of autistic parents, to enrich understanding of autistic lived experiences.

## Conclusion

Autistic adolescent girls face complex and diverse challenges in the school setting, specifically mental health issues, unmet social and education needs, and social exclusion. The purpose of this review was to provide an overview of research relating to the experiences of autistic females in secondary school settings by reporting on their experiences and the lived experiences of autistic women reflecting on their past. Based on the identified articles, the barriers girls face in the compulsory education setting centred on four themes of societal barriers grounded in gender; the institutional or physical barriers of schools; social and communicative expectations; and stigmatization. There is an overarching need to sensitize and educate widely on the topic of autism from teachers, to support staff, school psychologists, and peers of autistic youth. This should concentrate on the unique presentations of autism in girls and include the subsequent impact of missed or overlooked diagnoses has on the girls’ education experiences and outcomes. In addition, the results underscore the need for future research to centre diverse autistic lived experiences and knowledge. To address these barriers, autistic inclusion is needed at all levels of participation from self-advocacy and peer support spaces to the co-development of training and policy. Inclusion needs to represent diverse lived experiences and include marginalized subgroups within the autistic community.
